# “Is this blueberry ripe?”: a blueberry ripeness detection algorithm for use on picking robots

**DOI:** 10.3389/fpls.2023.1198650

**Published:** 2023-06-09

**Authors:** Yan Liu, Hongtao Zheng, Yonghua Zhang, Qiujie Zhang, Hongli Chen, Xueyong Xu, Gaoyang Wang

**Affiliations:** ^1^ School of Information and Electrical Engineering, Hangzhou City University, Hangzhou, China; ^2^ School of Information and Electronic Engineering, Zhejiang University, Hangzhou, China; ^3^ School of Computer Science and Technology, Zhejiang Sci-Tech University, Hangzhou, China; ^4^ School of Control Science and Engineering, Zhejiang University, Hangzhou, China

**Keywords:** vegetable, blueberries, YOLOv5x, MobileNetV3, Little-CBAM, MSSENet

## Abstract

Blueberries are grown worldwide because of their high nutritional value; however, manual picking is difficult, and expert pickers are scarce. To meet the real needs of the market, picking robots that can identify the ripeness of blueberries are increasingly being used to replace manual operators. However, they struggle to accurately identify the ripeness of blueberries because of the heavy shading between the fruits and the small size of the fruit. This makes it difficult to obtain sufficient information on characteristics; and the disturbances caused by environmental changes remain unsolved. Additionally, the picking robot has limited computational power for running complex algorithms. To address these issues, we propose a new YOLO-based algorithm to detect the ripeness of blueberry fruits. The algorithm improves the structure of YOLOv5x. We replaced the fully connected layer with a one-dimensional convolution and also replaced the high-latitude convolution with a null convolution based on the structure of CBAM, and finally obtained a lightweight CBAM structure with efficient attention-guiding capability (Little-CBAM), which we embedded into MobileNetv3 while replacing the original backbone structure with the improved MobileNetv3. We expanded the original three-layer neck path by one to create a larger-scale detection layer leading from the backbone network. We added a multi-scale fusion module to the channel attention mechanism to build a multi-method feature extractor (MSSENet) and then embedded the designed channel attention module into the head network, which can significantly enhance the feature representation capability of the small target detection network and the anti-interference capability of the algorithm. Considering that these improvements will significantly extend the training time of the algorithm, we used EIOU_Loss instead of CIOU_Loss, whereas the k-means++ algorithm was used to cluster the detection frames such that the generated predefined anchor frames are better adapted to the scale of the blueberries. The algorithm in this study achieved a final mAP of 78.3% on the PC terminal, which was 9% higher than that of YOLOv5x, and the FPS was 2.1 times higher than that of YOLOv5x. By translating the algorithm into a picking robot, the algorithm in this study ran at 47 FPS and achieved real-time detection well beyond that achieved manually.

## 1 Introduction

Blueberries are some fruit with high flavor and nutritional value and are loved all over the world, and as a result, blueberry cultivation is spreading rapidly around the globe (Li et al., 2018). Since the 21st century, China has been growing blueberries on a large scale and is the leading blueberry grower in the Asia-Pacific region (Li Y. D, et al., 2021). However, given the extreme difficulty and workload of blueberry picking, it has led to a significant reduction in efficiency. To address these issues, new automated picking robots are being created to fill the production gap. More and more detection algorithms are being created to enable these robots to have the ability to accurately identify the ripeness of the blueberries so that the robots can accurately pick the ripe fruit.

To alleviate manual pressure and labor costs while increasing the efficiency of ripeness detection, some traditional machine vision algorithms have been gradually introduced to detect fruit ripeness. For example, Aquino et al. (2017) used mathematical morphology with pixel classification to estimate the number of berries in a single cluster of grapevines, which has high stability, but the images run too slowly, which leads to a significant decrease in the real-time performance of the algorithm. Zhang et al. (2020) proposed a method based on multi-feature fusion with the support vector machine method for fruit counting with an accuracy of 78.15%. Liu et al. (2018) converted the image from RGB space to Y′CbCr space by applying a visual detection algorithm with an elliptic boundary model, and then introduced ordinary least squares (OLS) to fit an implicit second-order polynomial of the elliptic boundary model in Cr–Cb color space. Liu et al. (2019) proposed an apple fruit detection algorithm based on color and shape features with a recall of more than 85%. However, the robustness of the method is poor. Tan et al. (2018) explored a method to identify and count blueberry fruits based on different ripeness regions by applying the direction histogram of oriented gradient (HOG) features and color features to detect blueberry fruits, but this method had the problem of ineffective recognition of obscured fruits and took longer. Recently, ripening parameters of berries of wine and table grape cultivars have been predicted by using NIR devices with promising results (Ferrara et al., 2022a; Ferrara et al., 2022b), and these devices could be mounted on picking robots.

Taken together, these machine learning-based algorithms do have outstanding advantages over manual detection, but they still have the problem of low detection accuracy or slow detection speed. Given the realities of growing blueberries in clusters and the different maturity of each blueberry in each cluster, the complex environment in which blueberries are grown with serious background interference, and the fact that each blueberry is stuck together and obscured by the others. These situations can lead to traditional machine vision algorithms detecting the wrong ripeness of blueberries or failing to detect blueberries. Therefore, further attempts have been made to introduce deep learning-based algorithms to detect fruit ripeness (Tian et al., 2019; Cecotti et al., 2020; Kuznetsova et al., 2020; Aguiar et al., 2021; Li H, et al., 2021; Lu et al., 2021; Wu et al., 2021; Zheng et al., 2021; Hou et al., 2022; Li et al., 2022; Zheng et al., 2022). For example, Zhu et al. (2020) proposed a faster R-CNN-based algorithm for blueberry fruit detection and recognition that was able to identify blueberries and distinguish their ripeness more accurately and quickly under sunny conditions, but the algorithm was greatly influenced by either the background or light and was not robust. Yang et al. (2022) proposed a lightweight blueberry fruit detection algorithm for multi-scale targets that incorporates a novel attention mechanism. MacEachern et al. (2023) applied YOLOv4 to blueberry ripeness detection and showed that the algorithm has high accuracy for blueberry ripeness detection but given the large computational effort of the YOLOv4 model, later migration to a small, embedded device would lead to a significant reduction in algorithm speed. These deep learning-based algorithms represent a quantum leap in both accuracy and speed compared to traditional machine vision algorithms. However, they are not perfect, and if installed on a platform with good computing power, they can show their performance advantages, but they still have a large number of parameters, which makes it impossible to run these algorithms on some low-power embedded platforms. Manufacturers of agricultural automation equipment currently do not install computationally powerful graphics cards or embedded devices on their equipment in order to reduce the cost of manufacturing the equipment. Therefore, considering the application prospects of our algorithm, we need to reduce the amount of computation required during the algorithm’s operation while improving its accuracy, thus making the algorithm less demanding on the device’s computational power. At the same time, there are many uncertainties in the field, such as changes in light, cultivar growing habits and trellising systems, weather, and air visibility, which can affect the algorithm’s accuracy, so we also want the algorithm to be resistant to these objective factors.

## 2 Materials and methods

### 2.1 Algorithm design

**Figure 1:**
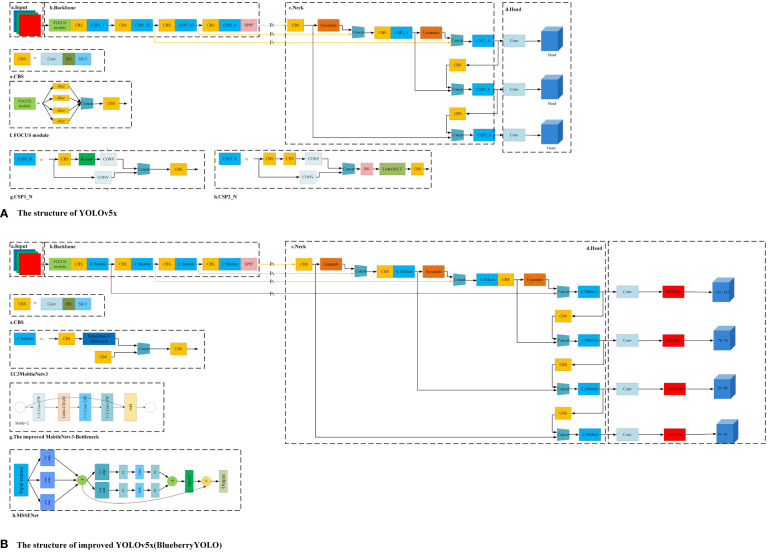
Structural diagram of YOLOv5x and the algorithms in this paper. Where **(A)** represents the network structure of YOLOv5x and **(B)** represents the network structure of the improved BlueberryYOLO.

To achieve the lightweightness of the blueberry detection algorithm and improve detection accuracy, this paper makes various improvements based on the algorithm structure of YOLOv5x. The final improved algorithm structure is shown in **Figure 1B**, where **Figure 1A** shows the YOLOv5x algorithm. To facilitate the description, we divide the algorithm into three parts: backbone structure, neck structure, and head structure, and the specific improvements are as follows:

1. For the backbone network part, the structure of MobileNetv3 (Howard et al., 2019) is used in this paper instead, considering that the original structure of YOLOv5x is complex and computationally expensive. MobileNetv3 uses a variety of lightweight strategies and is able to greatly reduce the computational cost of the algorithm. However, after replacing the original backbone network with MobileNetv3, the overall algorithm has shown a certain decrease in its ability to focus on and extract valid information. Therefore, this paper continues to incorporate a lightweight attention mechanism called Little-CBAM into the MobileNetv3 structure. Little-CBAM is an improvement of the CBAM (Woo et al., 2018) structure that enhances the network’s ability to integrate channel and spatial information and adjusts the attention weights of target regions.
2. For the neck network, the original YOLOv5x of this part was used eight times, 16 times, and 32 times downsampling for the backbone network to obtain the feature layers of P3, P4, and P5, respectively. The three lines above are shown in **Figures 1A, B**. However, if the downsampling times are too large, then the deeper feature maps in the model training process will not be able to learn the feature information of the small-sized blueberries. To solve this problem, we add a four-time downsampling feature layer P2 between the backbone network and the neck network, as shown by a red line in **Figure 1B**.
3. For the head structure of YOLOv5x, this paper embeds an improved SENet (Hu et al., 2018) attention mechanism (MSSENet). This structure can enhance feature representation in small target detection networks and anti-interference capability in complex contexts.

After making these changes to the algorithm’s backbone, neck, and head, the algorithm took longer to train, and the loss curve did not fit for a long time. To solve these problems, the following scheme is proposed in this paper:

1. For the algorithmic loss function, the Efficient Crossover Joint Loss (EIOU_Loss) function is used to optimize the model’s overall performance. Moreover, this loss function can reduce the model training time and improve the final detection accuracy compared with the original loss function.
2. We use the K-means++ algorithm (Arthur and Vassilvitskii, 2006) to cluster the dataset used for algorithm training so that the generated pre-defined anchor frames are more adapted to the scale size of blueberries, thus improving the accuracy of target detection. In addition, it also reduces the time required for model training.

### 2.2 Dataset collection

The aim of this study was to identify the ripeness of blueberries grown in a natural environment, which is not only complex but also highly susceptible to severe disturbances by either internal factors (leaves, branching, size of clusters, etc.) or external factors (light, climatic conditions, etc.) (MacEachern et al., 2023). Second, blueberry fruits are closely adhered to each other, and each cluster usually contains blueberries of different maturity levels.

**Figure 2:**
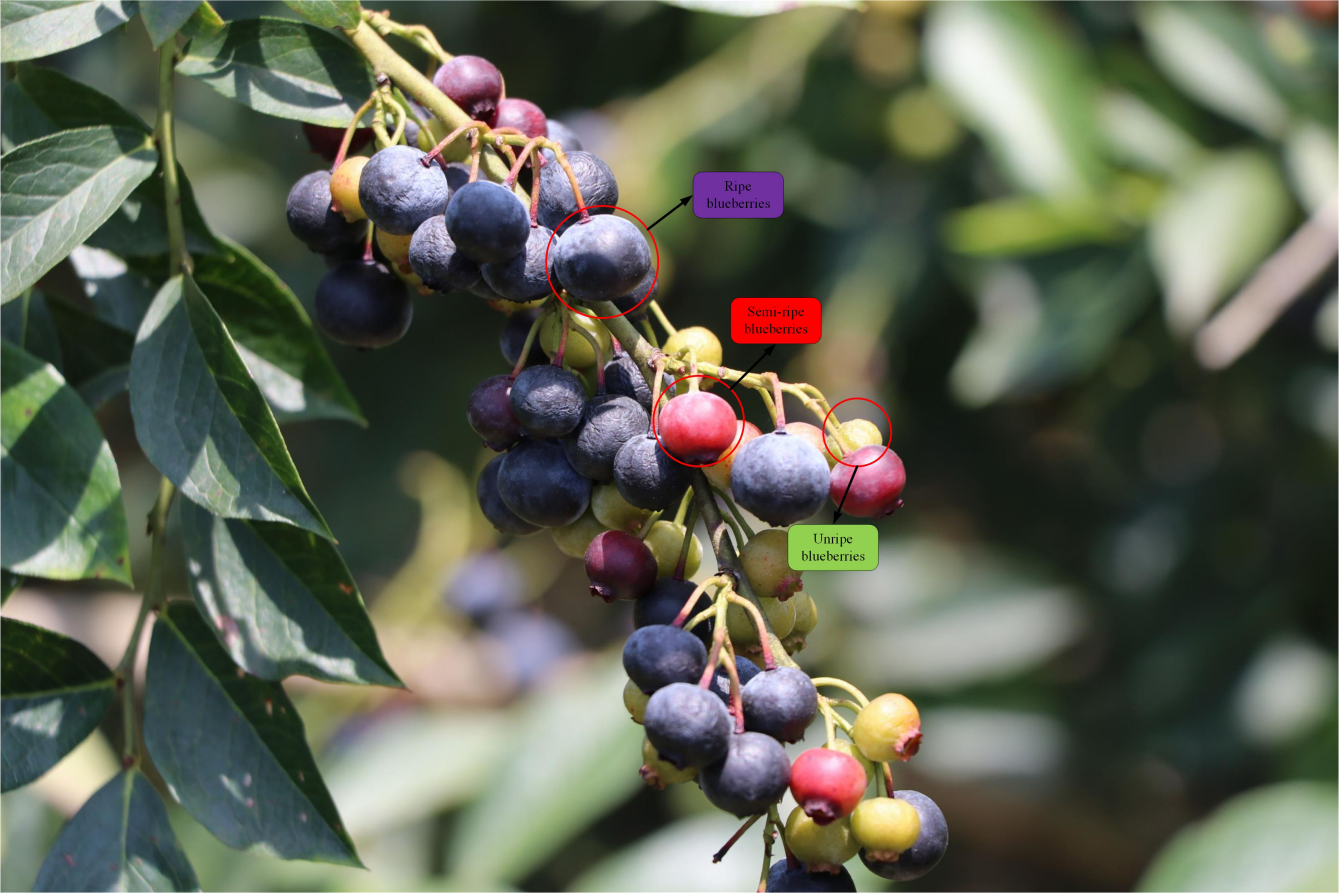
Diagram of the three ripeness levels of blueberries.

Since this paper focuses on analyzing the application of computer vision algorithms to blueberries, the vision algorithms are distinguished based on the differences in the appearance characteristics of different ripe blueberries, such as color shades, hue saturation, and shape size. Based on these features, we distinguished blueberries into the following three categories: ripe, semi-ripe, and unripe, as shown in **Figure 2**. Ripe blueberry color is dark purple, large volume, overall color is dark, sugar content is about 15%, acidity is about pH 4.35, TSS is 12.1%; semi-ripe blueberry color is red or lavender, small volume, bright color, sugar content is about 9%, acidity is pH 3.95, TSS is 8.1%; immature blueberry color is green, small volume, very bright color. The unripe blueberries were green in color, small in size, and very bright in color, with a sugar content of 3.8%, an acidity of pH 3.6, and a TSS of 6.8%. In addition, the central role of the algorithm in this paper is to identify the different ripeness of blueberries by the changes in color, morphology, and volume.

The dataset used in this paper was expanded from the published dataset in Reference (Li et al., 2022), with a total of 9,312 images. The dataset was taken at Prince Blueberry Estate in Xinjian District, Nanchang City, Jiangxi Province, with a cultivar of high irrigation blueberry and a planting density of about 3,200 plants per hectare, with a plant spacing of 1.0–1.2 m and a row spacing of 2.5 m. The annual yield of each plant was about 1.5–2.5 kg.

### 2.3 Dataset annotation and processing

The images are annotated using the annotation tool labeling in the format of the Pascal VOC dataset to produce an.xml annotation file. Deep learning algorithms require a large amount of data to achieve good detection results, and too few training images can lead to overfitting of the model, so this paper performs data enhancement operations on the 9,312 raw photos generated from direct camera shots in *Section 2.1*. The data enhancement operations we employ specifically include flipping, scaling, panning, rotating, adding random noise combinations, and so on, and simultaneously transforming the corresponding annotation files for each image. The data sets were randomly divided into training (18,498 images), validation (2,642 images), and test (5,286 images). The distribution of the dataset by type is shown in **Table 1**. In this table, we highlight several factors that can interfere with the target detection algorithm, including light intensity, the density of fruit distribution on each blueberry tree, and the clarity of the camera when capturing images, among others. In the category of light intensity, Backlighting indicates that there is no direct sunlight on the fruit, Normal indicates that the light on the fruit is soft and non-irritating, Strong indicates that the fruit is exposed to direct light and the light intensity is strong. In the category of fruit density, Very sparse indicates that the fruit is scattered, and Normal indicates that the distribution of fruits is very tight, but the phenomenon that adjacent fruits do not obscure each other is not severe. Tightly arranged indicates that the distribution of fruits is tight, but the phenomenon that adjacent fruits obscure each other is very serious. In the category of fruit shot clarity, Very blurry indicates that the blueberry image in the camera is very blurred. Partially blurred indicates that the blueberry image in the camera is partially blurred but not deep, and Very clear indicates that the blueberry image in the camera is very clear.

**Table 1:** The quantities in each type of dataset.

Types	Number	Light intensity				Fruit density		Fruit shot clarity		
		Backlighting	Normal	Strong	Very sparse	Normal	Tightly arranged	Very blurry	Partially blurred	Very clear
Training	18,498	3,453	9,576	5,469	2,878	9,945	5,675	2,676	8,767	7,055
Validation	2,642	756	1,323	563	645	1,565	432	544	1,543	555
Test	5,286	1,232	3,445	609	531	4210	545	756	3,879	651

### 2.4 Training and experimental comparison platform

The hardware and software used in this experiment were as follows: Ubuntu 18.04, NVIDIA GeForce RTX 3070; operating system: CentOS 7.6; CUDA version 11.2, CUDNN8.2.1, OpenCV version 4.5.3, and deep learning framework: TensorFlow 2.5.

### 2.5 Platform for practical application of algorithms

**Figure 3:**
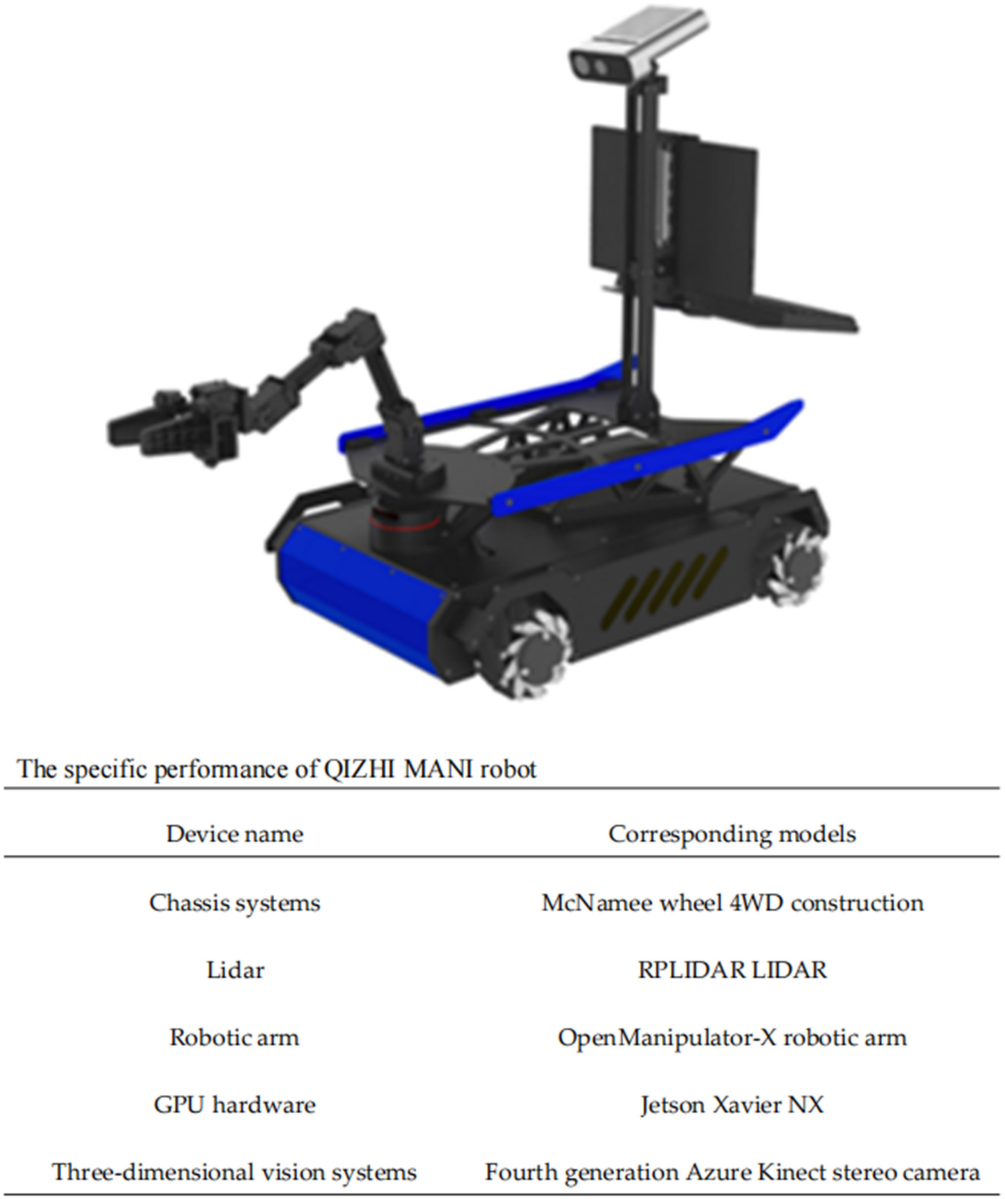
Appearance of the picking robot and types of accessories.

The picking robot used in the algorithm in this study has the appearance and the various components on this robot are shown in **Figure 3**. The computing platform of the picking robot is a Jeston Xavier NX, an embedded platform with many times less computing power than the RTX3070 presented in *Section 2.3*.

## 3 Algorithm structure analysis

### 3.1 Introducing Little-CBAM to MobileNetv3

Compared with the YOLO (Redmon and Farhadi, 2017; Redmon and Farhadi, 2018) model, YOLOv5x has improved speed and accuracy, but the model is still significant and unsuitable for deployment on embedded devices with small video memory and arms. Therefore, we replaced the modules CSP1_N and CSP2_N in **Figure 1A**a with C3Mobile. The structure of C3Mobile is shown in **Figure 1B**f, where the MobileNetv3-Bottleneck is the basic unit that constitutes MobileNetv3.

MobileNetv3-Bottleneck uses deeply separable convolution. The number of model parameters is significantly reduced compared to conventional convolution without compromising the model’s performance. The ratio of the decrease in the number of parameters is:


(1) $$\frac{D_{in}\times k\times k+D_{in}\times D_{out}}{D_{in}\times k\times k\times D_{out}}=\frac{1}{D_{out}}+\frac{1}{k^{2}}$$

However, considering the complex background of the actual blueberry plantation, the accuracy of blueberry detection will be reduced to some extent after the YOLOv5x backbone network is changed to MobileNetv3; therefore, we embed Little-CBAM on top of the MobileNetv3 structure. The structure of the specific modified MobileNetv3 is shown in **Figure 1B**g. Little-CBAM was obtained by improving CBAM. CBAM is an attention algorithm that uses a fully connected layer to map the tandem channels and spaces of the feature map, and the fully connected layer is a computationally resource-intensive method. In contrast, using 7 × 7 convolutional kernels for spatial feature extraction in spatial attention enhances the perceptual field but also increases computational effort.

**Figure 4:**
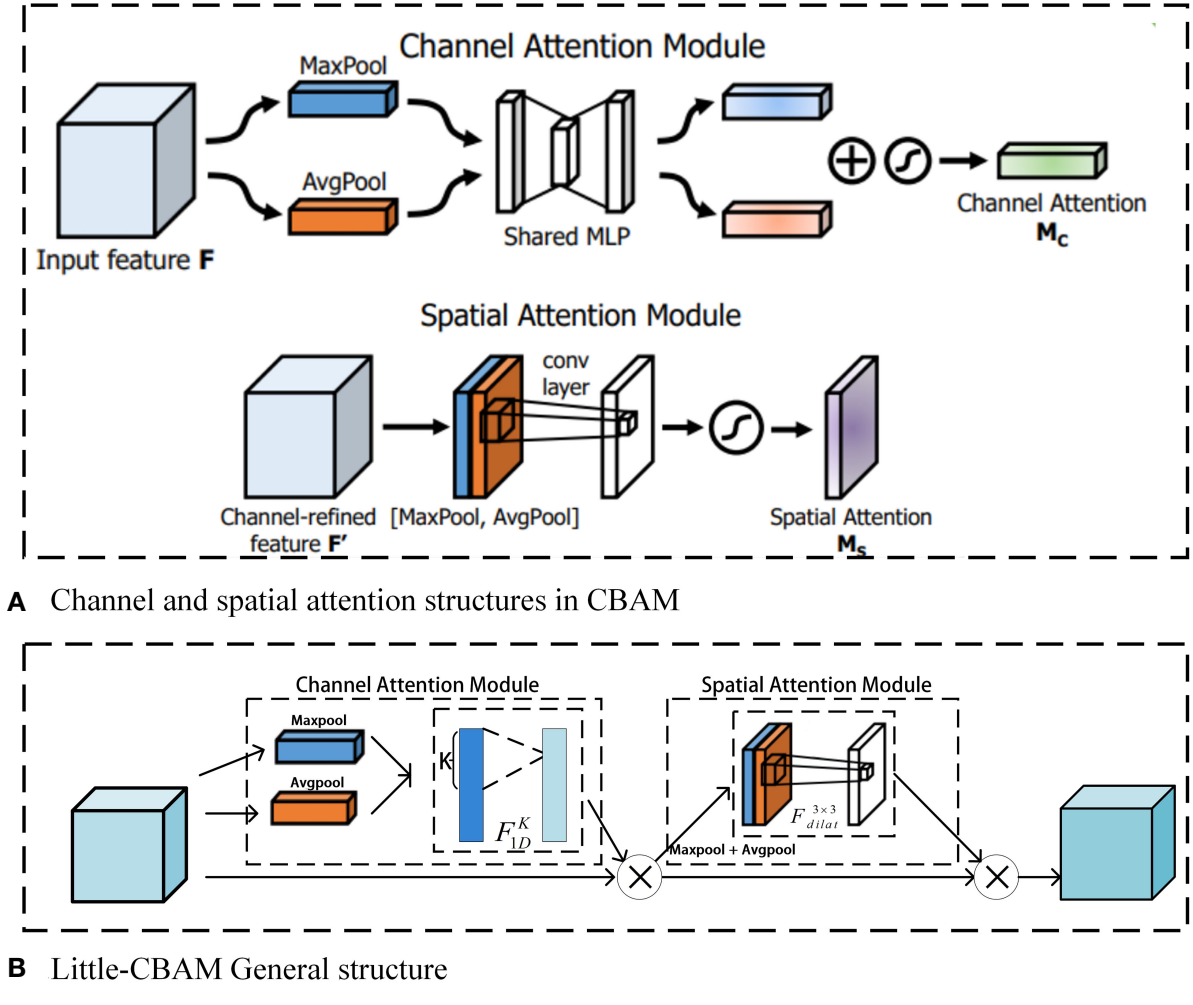
Structure of CBAM and improved CBAM (Little-CBAM). Where **(A)** is the network structure of CBAM and **(B)** is the network structure of the improved Little-CBAM.

In the ECA algorithm, the closer the channels are to each other, the higher the correlation between them; therefore, using a convolution with a one-dimensional kernel instead of a fully connected layer can significantly reduce the computational parameters. In the literature (Yu et al., 2017), a null convolution is proposed, which replaces the 7 × 7 convolution with a 3 × 3 null convolution with a null rate of only 2. The final perceptual field was the same, but the number of parameters was only 9/49 of the original one. To achieve this effect, CBAM combines the above two points and significantly reduces the number of parameters without reducing accuracy. **Figure 4** contains CBAM and the improved Little-CBAM, where **Figure 4A** is the network structure of CBAM. The improved structure is shown in **Figure 4B**, where $F_{1D}^{K}$ denotes a one-dimensional convolution with a convolution kernel of k, and $F_{dilat}^{3\times 3}$ denotes a 3 × 3 convolution with a void rate of 2.

The insertion of Little-CBAM into MobileNetv3-Bottleneck introduces only a small number of parameters, but it enhances the network’s ability to integrate channel and spatial information and adjust the attention weights of the target region. Specifically, for the MobileNetv3-Bottleneck, the first Ghost module expands the number of channels, after which the insertion of Little-CBAM is the most cost-effective.

### 3.2 Improved neck

**Figure 5:**
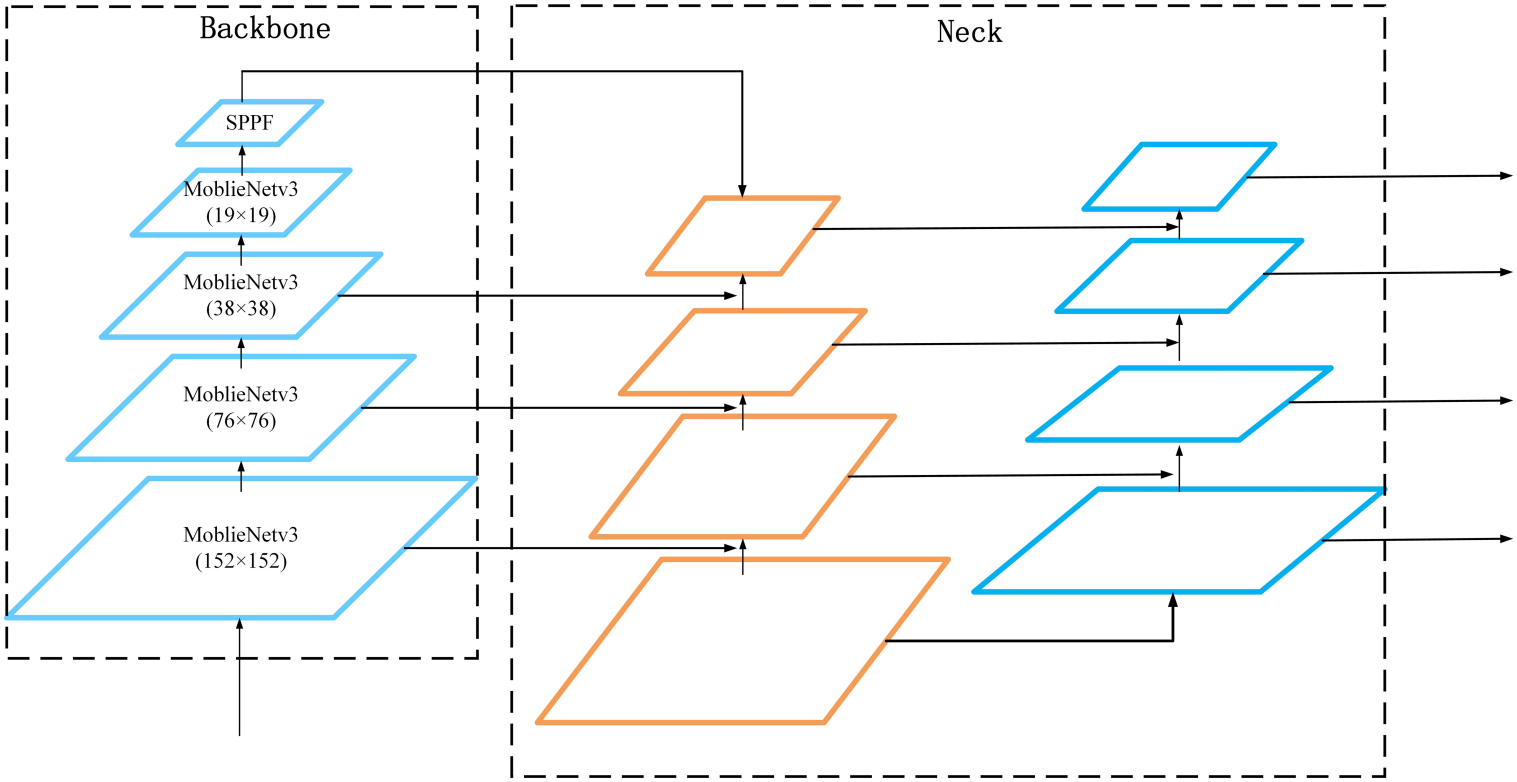
Improved neck structure of the YOLOv5x.

Considering that blueberries appear entirely in bunches, the number of targets to be detected in a single image is large, and the scale of color variability is high. The original YOLOv5x model was poor at detecting small targets because of the loss of small-target information during the convolution and downsampling processes. This study improved the sensitivity of the model to small targets by expanding the shallow structure of the YOLOv5x backbone by one layer and adding a new detection head at the detection end. **Figure 5** shows the improved neck structure. It consists of adding a convolutional layer and upsampling after the 76 × 76 feature layer, and then fusing the two upsampling feature layers with the 152 × 152 feature layer to obtain a 152 × 152 detection feature map for small target detection.

### 3.3 Introduction and analysis of the MSSENet structure

In this study, we designed SENet to generate a multi-scale fused attention module (MSSENet) by constructing multi-scale fused features and a multi-method feature extractor to enhance feature representation in small target detection networks and prevent interference from complex backgrounds. The structure of MSSENet is inspired by (Zhu et al. 2022).

The next step of feature extraction processing is first performed by integrating multiple scales of feature maps through different scales of convolution kernels. The multi-scale feature fusion within the convolutional layer allows the output feature map to combine resolution and semantic information, providing a richer feature set for the subsequent feature extractor.

The squeezing operation of SENet was subsequently improved to obtain adaptive channel feature information. SENet was designed to construct a feature extractor to obtain global features through global averaging pooling operations. For small targets with little feature information, focusing only on global features may result in a loss of feature information, whereas the global maximum pooling operation focuses more on local features. Therefore, this study introduces two pooling methods for feature extraction that can better capture the local feature information of small targets and enhance the feature extraction capability of the attention mechanism in complex contexts.

Finally, the adaptive channel feature information generated by the two pooling methods is summed and activated to generate attention weight information, which is then weighted with the input feature map to obtain the channel attention map. The Mish (Misra, 2019) activation function is used instead of the ReLU activation function for the first full-connected dimensionality reduction operation, which avoids the use of the ReLU activation function to sparse out too many effective features and helps the module obtain more non-linear relationships. The improved MSSENet attention module is shown in **Figure 1Bh**, where “+” represents features for summing operations and “×” represents features for weighting operations. For the input feature *X ∈ R^c × h × w^*, *c* is the number of input channels, *h* is the input height, and *w* is the input width. Then the operation of extracting features by convolution of different sizes for the input features is


(2) $$X_{c}=v_{3\times 3}X+v_{5\times 5}X+v_{7\times 7}X$$

where *V* is the convolution of the input features using different-sized convolution kernels, and *Xc* is the output features convolved with different-sized convolution kernels.

Two separate pooling operations are then performed to obtain better channel feature information, with the original multi-scale features from the previous step as input.


(3) $$X_{avg}=\frac{1}{h\times w}\sum_{i=1}^{h}\sum_{j=1}^{w}X_{c}(i,j)$$

(4) $${\text{X}}^{\text{max}}\;=\text{MAX}({\text{X}}^{\text{c}}(\text{i},\text{j}))$$

where: $X_{avg}$ is the global average pooling operation and $X_{\max}$ is the global maximum pooling operation.

The channel attention vectors are generated by the following equations: Xa for feature extraction on the global average pooling branch and Xm for feature extraction on the global maximum pooling branch. As the features were extracted, they were transformed into a nonlinear space to complete the activation operation.


(6) $$X_{a}=FC_{2}(Mish(FC_{1}(X_{avg})))$$

(7) $$X_{m}=FC_{2}(Mish(FC_{1}(X_{\max})))$$

(8) $$X_{s}=Sigmoid(X_{a}+X_{m})$$

where: the input is the multi-scale fusion feature from the previous step; Sigmoid is the normalization function; FC is the fully connected function; and Mish is the non-linear activation function.

Next, the calculated attention weights are weighted to the feature map generated in the first step as the final channel attention feature map.


(9) $$X_{weight}=Scale(X_{c},X_{s})$$

### 3.4 The improved loss fuction

In this study, CIOU_Loss of the original model was replaced by EIOU_Loss with a more accurate evaluation and faster convergence, which can improve the overall performance of the model and compensate to a certain extent for the increase in training time and slower convergence caused by the addition of the multiscale feature fusion module.

CIOU_Loss considers the overlap area, centroid distance, and aspect ratio of the bounding box regression but ignores the true difference between the width and height and their confidence levels, which hinders the effectiveness of the model optimization. The EIOU_Loss penalty term used in this study contains three components: the overlap loss LIOU, the centroid distance loss L_dis_, and the aspect loss L_asp_. The first two adopt the advantages of the method in CIOU_Loss, whereas the aspect loss directly sets the optimization target to the minimum difference between the width and height of the real and predicted boxes, resulting in faster convergence. The formula is


(10) $$L_{EIOU}=L_{IOU}+L_{dis}+L_{asp}=1-I+\frac{\rho^{2}(b,b^{gt})}{c^{2}}+\frac{\rho^{2}(w,w^{gt})}{c_{w}^{2}}+\frac{\rho^{2}(h,h^{gt})}{c_{h}^{2}}$$

where: $c_{w}$ and $c_{h}$ are the width and height of the minimum external frame covering the real frame and the predicted frame.

### 3.5 Using K-means++ clustering algorithm

The original anchor frame size of the YOLOv5x model is based on the clustering of the labeled target frame of the COCO dataset, which is different from the fault target size of the transmission line; therefore, the direct use of the model affects the detection performance of the model. The steps are as follows:

(1) A randomly selected sample target frame is used as the initial cluster center, and the minimum intersection over union (IOU) distance A(x) between the remaining sample frames and the current cluster center is calculated as


(11) $$A(x)=1-I_{\left(x,c\right)}$$

(2) The probability O(x) of each insulator sample frame being selected as the next cluster center is calculated, and the next cluster center is selected using the roulette wheel method.


(12) $$O(x)=\frac{A{\left(x\right)}^{2}}{\sum_{x\in X}A{\left(x\right)}^{2}}$$

where X is the total sample of target marker frames.

(3) Steps (1) and (2) are repeated until K clusters are selected.

(4) The distance from each sample x to the K cluster centers in the dataset is calculated, the sample is assigned to the class corresponding to the cluster center with the smallest distance, the cluster centers for each class are re-calculated as shown in Equation (13), and the update of the classification and cluster centers is repeated until the anchor box size remains the same.


(13) $$c_{l}=\frac{1}{\left|c_{l}\right|}\sum_{x\in c_{l}}x$$

where: l = 1, ····, K; K is the number of anchor frames of different sizes, the value of which is determined by the number of anchor frames in the detection model. Since the detection model in this paper contains four inspection feature maps, each of which corresponds to three anchor frames.

After anchor frame optimization, the models of the four inspection heads correspond to the 152 × 152, 76 × 76, 38 × 38 and 19 × 19 feature maps and corresponding anchor frames in **Table 2**.

**Table 2:** The corresponding anchor frames.

Types of targets	152 ×152	76 ×76	38 ×38	19×19
**Ripe blueberries**	(13,39)	(35,53)	(120,19)	(276,56)
	(13,44)	(41,60)	(181,39)	(272,104)
	(46,9)	(65,46)	(54,157)	(399,82)
**Semi-ripe blueberries**	(11,33)	(38,51)	(125,34)	(265,53)
	(17,32)	(42,55)	(167,45)	(261,113)
	(40,13)	(73,45)	(67,134)	(387,113)
**Unripe blueberries**	(15,24)	(41,49)	(132,25)	(259,67)
	(22,29)	(52,60)	(155,51)	(249,153)
	(29,15)	(75,46)	(87,123)	(369,134)

## 4 Evaluation indicators

In this study, the performance of the target detection model was verified using the metrics of precision (P), recall (R), mean average precision (mAP), and frames per second (FPS), expressed as follows:


(14) $$P=\frac{TP}{TP+FP}$$

(15) $$R=\frac{TP}{FN+TP}$$

(16) $$AP=\int_{0}^{1}P(r)dr$$

(17) $$mAP=\frac{\sum_{i=1}^{k}AP_{i}}{N}$$

(18) $$FPS=\frac{1}{t_{avg}}$$

where TP and FP are the numbers of accurately and incorrectly identified samples, respectively; FN is the number of unidentified samples; accuracy P is the probability that a detected sample is correctly predicted; recall R denotes the probability that a certain category of samples is detected; N denotes the number of categories set by the model; N = 3 corresponds to the three maturity levels of blueberries; and *t_avg_* is the average time required to detect a picture.

The mAP is the average accuracy of the algorithm in identifying blueberries of three maturity levels, which can represent the comprehensive accuracy of the algorithm, so in the subsequent comparison experiments we mainly compare the magnitude of this parameter to derive the strength of the comprehensive accuracy of the algorithm.

## 5 Results and discussion

### 5.1 Ablation experiments

The YOLOv5x algorithm was benchmarked by adding each of the improvements mentioned in the study to evaluate the contribution of each improvement to mAP and real-time performance. Tests were conducted using a test set from the dataset used in this study.

From **Table 3**, Experiment 2 shows a more than twofold increase in speed compared to Experiment 1 after using the MobileNetv3 lightweight network as the backbone feature extraction network, at the cost of an 86% decrease in accuracy. Experiment 5 showed a slight decrease in accuracy compared to Experiment 1 but a significant increase in speed, which suggests that we have achieved a lightweighting of the algorithm. Experiment 3 showed no significant decrease in speed compared to Experiment 1 after adding an extra layer to the neck structure of the algorithm, but at the same time a more significant increase in accuracy. Compared with Experiment 1, Experiment 4 showed that the overall speed of the algorithm did not decrease significantly after MSSENet was embedded in the head network; however, the recognition accuracy improved significantly. Experiment 13 is the proposed algorithm, which has a significant improvement in both accuracy and speed compared to Experiment 1. This shows that the improvements in our algorithm have had a significant effect to some extent.

**Table 3:** Ablation Experiments for YOLOv5x.

Methods	MobileNetv3	Little-CBAM	Improved Neck	MSSENet	mAP (%)	FPS
1. YOLOv5x					69.3%	40.41
2	✓				58.1%	99.54
3			✓		71.3%	35.12
4				✓	72.1%	35.45
5	✓	✓			65.6%	95.13
6	✓		✓		62.5%	94.14
7	✓			✓	65.3%	95.43
8			✓	✓	75.4%	27.54
9	✓		✓	✓	65.8%	88.39
10	✓	✓		✓	71.2%	86.43
11	✓	✓	✓		76.5%	89.44
12. BlueberryYOLO	✓	✓	✓	✓	78.4%	83.64

Where “✓” symbol represents the improvement of the structure of the ordinate corresponding to this symbol.

Compared with **Table 4**, the computation and model size of this algorithm are only one-sixth of YOLOv5x and are close to YOLOv5s, which achieves light weighting of the algorithm; however, the accuracy is significantly better than that of other algorithms.

**Table 4:** Performance comparison of different networks.

Type of algorithm	mAP (%)	R (%)	FPS	FLOPS	Params (MB)
YOLOv5x	67.3%	68.2%	40.41	205.7	86.7
YOLOv5s	53.1%	54.8%	89.75	16.5	7.2
YOLOv5m	58.1%	59.4%	71.84	49.0	21.2
YOLOv5l	64.5%	65.1%	60.63	109.3	46.5
BlueberryYOLO	78.3%	75.9%	83.64	31.3	13.1

### 5.2 Ablation experiments

To verify the effectiveness of the backbone network lightweighting combined with attention mechanism improvement, comparison experiments were conducted to embed different attention mechanisms in the backbone network. **Table 5** shows the final experimental results. In Experiments 2 and 3, we found that mAP, R, and P improved to a certain extent, but FPS decreased significantly, which proves that the idea of embedding the attention mechanism in MobileNetv3 is valid. By comparing Experiments 2 and 4, we found that mAP, R, and P improved to a certain extent, and FPS only decreased slightly, indicating that embedding a small amount of CBAM in MobileNetv3 can significantly improve the accuracy of the algorithm while maintaining its speed. By comparing Experiments 3 and 4, we found that the mAP, R, and P of the two algorithms were essentially the same, but with a significant speed-up. By comparing Experiments 1 and 4, we found that the modified algorithm has only a slight decrease in accuracy and a significant increase in speed, which initially demonstrates the effectiveness of embedding Little-CBAM in MobileNetv3.

**Table 5:** Different attention mechanisms embedded in the backbone network.

Types of algorithms	mAP (%)	R (%)	P (%)	FPS
1. YOLOv5x	69.3%	68.2%	71.3%	40.41
2. +MobileNetv3	58.1%	61.3%	59.6%	99.54
3. +MobileNetv3+CBAM	66.1%	68.3%	67.3%	80.43
4. +MobileNetv3+Little-CBAM	65.6%	67.4%	66.6%	95.13

### 5.3 Verifying the role of MSSENet

To verify the superior performance of the MSSENet proposed in this study, we added it to the head structure of YOLOv5x separately from other commonly used attention mechanisms. The final experimental results are listed in **Table 6**, from which we can observe that the values of P, mAP, and R for the MSSENet structure embedded in the head network significantly exceed the effects of embedding other attention mechanisms. This phenomenon indicates that the MSSENet proposed in this study has a considerably higher accuracy improvement for blueberry detection than the other attention mechanisms.

**Table 6:** Head networks embedded in multiple attention mechanisms.

Attention mechanisms added	P (%)	mAP (%)	R (%)
Not added	71.3%	69.3%	68.2%
+SENet	72.3%	70.3%	69.1%
+CBAM (Woo et al., 2018)	72.5%	69.6%	69.2%
+ECA (Wang et al., 2020)	73.1%	71.1%	70.2%
+CA (Hou et al., 2021)	72.5%	70.1%	69.2%
+SOCA (Dai et al., 2019)	73.3%	70.4%	69.3%
+MSSENet	74.4%	72.1%	71.4%

### 5.4 Validity of loss function improvements

Two different models were used to train and detect blueberry images, where Model A was BlueberryYOLO with a loss function of CIOU_Loss and Model B was BlueberryYOLO with a loss function of EIOU_Loss. The initial learning rate was set to 0.001, and the weight was decreased to 0.0005. The training process lasted for approximately 2,000 batches (epochs), and stochastic gradient descent was used as an optimization function to train the models.

**Figure 6:**
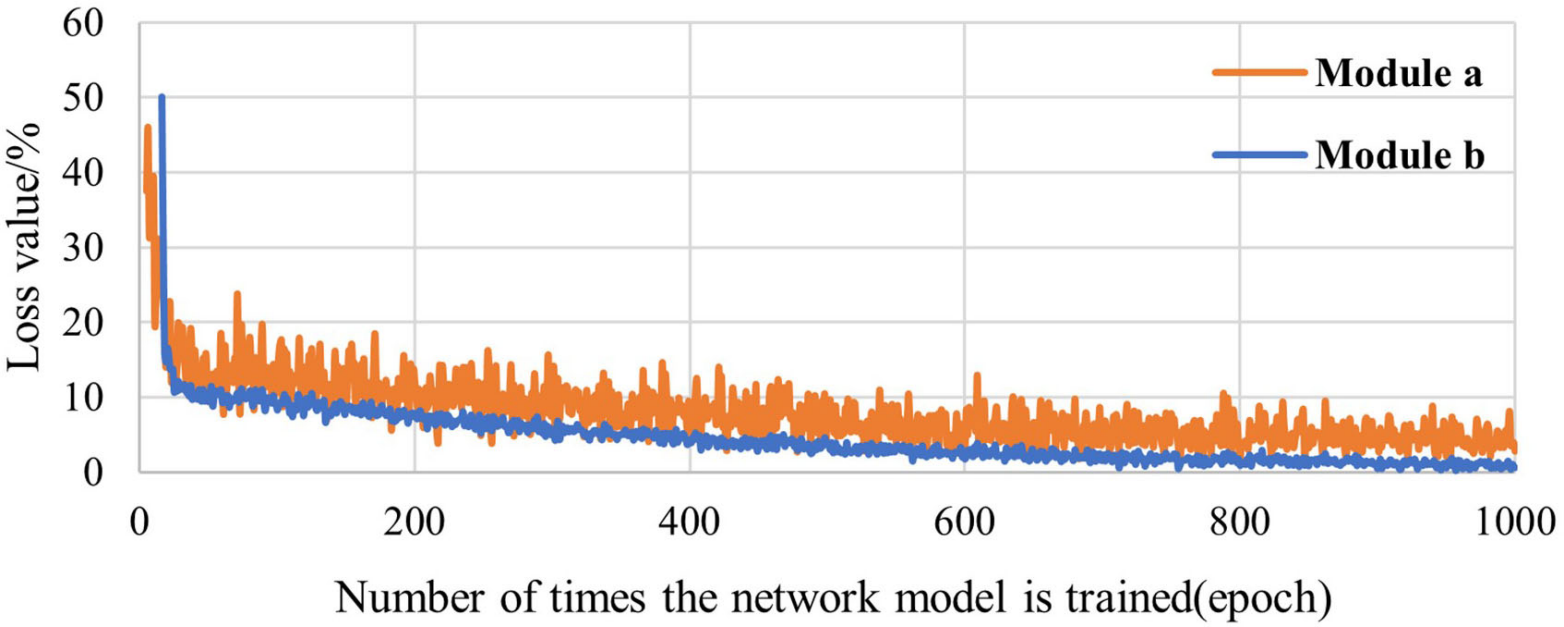
Loss value curves of different comparison models.

Considering that the role of the loss function in the training process of different algorithms is mainly in the early stages of model training, we focused on the loss value curves of the first 1,000 batches. A comparison of the loss value curves of different models is shown in **Figure 6**. Model A is in a constant state with large oscillations. Compared to Model A, Model B has a larger initial loss value than Model A owing to the EIOU_Loss splitting the width and height losses. However, Model B shows a better decrease rate and convergence ability than Model A after approximately 50 iterations, which confirms that the EIOU_Loss helps accelerate the decrease rate and shorten the convergence time of the model. The final loss value of Model B after convergence was 2.12%, which is significantly lower than that of Model A. This demonstrates that the model with the adjusted loss function can achieve improved training and detection results.

### 5.5 Comparison of detection performance of different algorithms

We have demonstrated the effectiveness and advantages of each part of the proposed algorithm in *Sections 5.1–5.4*, and in this section we demonstrate the overall performance of our algorithm by comparing it with other popular and advanced target detection algorithms to demonstrate the comprehensive performance of our algorithm. The data are listed in **Table 7**, from which we can conclude that the accuracy of Algorithm 7 is higher than that of all the other algorithms. In terms of speed, Algorithm 7 was slightly slower than Algorithm 5, but Algorithm 5 was significantly less accurate than Algorithm 7. Therefore, for balance, the overall performance of Algorithm 7 is significantly better than that of Algorithm 5.

**Table 7:** Comprehensive performance comparison of multiple algorithms.

Method	mAP (%)	R (%)	P (%)	FPS
1. Faster R-CNN (Ren et al., 2015)	59.9%	61.4%	53.4%	8.11
2. SSD (Liu et al., 2016)	58.7%	54.7%	61.4%	26.71
3. YOLOv3	69.3%	65.8%	72.1%	22.61
4. YOLOv4 (Bochkovskiy et al., 2020)	74.5%	72.3%	76.5%	20.13
5. YOLOv4-Tiny	52.1%	56.1%	49.4%	108
6. YOLOv5x	69.3%	68.2%	71.3%	40
7. BlueberryYOLO	78.3%	75.9%	79.3%	83.64

**Figure 7:**
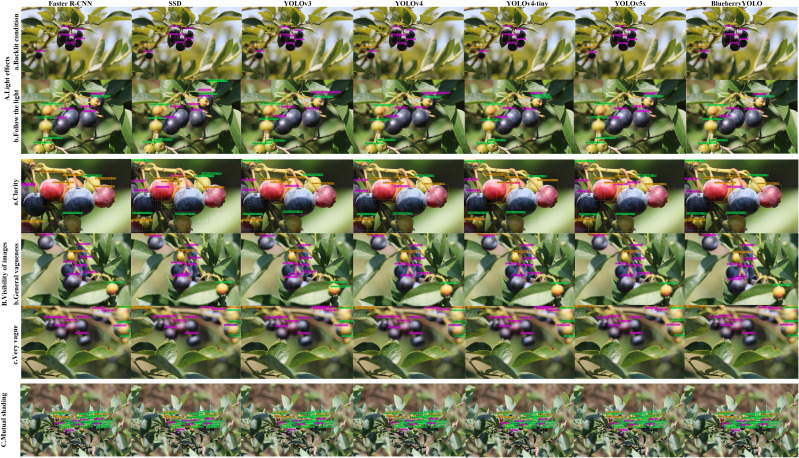
Multiple algorithms for immunity to environmental changes. **(A)** represents multiple algorithms for immunity to environmental changes. Aa represents backlighting and Ab represents front lighting. **(B)** represents multiple algorithms for blueberry image clarity immunity. Ba represents clear lighting, Bb represents general blurring, and Bc represents severe blurring. **(C)** represents multiple algorithms for immunity to interference between fruits when there is severe occlusion between them.

The proposed algorithm was primarily used for blueberry ripeness detection in outdoor fields, and the strength of the algorithm’s immunity to changes in objective factors, such as the environment, must be considered. This section demonstrates the immunity of the proposed algorithm to changes in three objective factors: changes in light, the sharpness of the photographed fruit, and fruit density. The test results are presented in **Figure 7**. From **Figure 7A**, the other algorithms have more or less false or missed detections, but the proposed algorithm does not, and each detection result has a high confidence level. From **Figure 7B**, the proposed algorithm can also effectively apply the interference caused by image sharpness, and the detection results are generally much better than those of the other algorithms. In **Figure 7C**, the proposed algorithm shows the best resistance to interference when dealing with mutual occlusion between blueberries. The above analysis shows that the algorithm can reasonably cope with interference caused by environmental changes.

### 5.6 Validation of practicality

The above experimental results on a PC suggest that the proposed algorithm has considerably good application potential. However, experiments are required to verify whether the algorithms are stable on a platform with low computing power, such as a picking robot. The main control computer of the QIZHI MANI robot uses the new Jetson Xavier NX computing unit from NVIDIA.

The final experimental results are shown in **Table 8**. +TensorRT appearing in the table refers to the application of the algorithm to the TensorRT framework for acceleration. The main role of the TensorRT framework is to accelerate the algorithm by significantly invoking the computational power of the hardware while essentially not losing the accuracy of the algorithm. All experiments in *Section 5.6* apply the TensorRT framework acceleration to the YOLO family of algorithms (YOLOv3, YOLOv4, YOLOv4-Tiny, YOLOv5x, and BlueberryYOLO). We find that the mAP, R, and P in this table are only slightly decreased compared to **Table 7**. This shows that the migration of the algorithm in this paper to the picking robot does not affect the accuracy of the algorithm. We continue to observe the FPS of the seven algorithms and find that the algorithm in this paper runs at a frame rate of 47 FPS, which indicates that our algorithm is able to run quickly on the picking robot. We find that the mAP, R, and P in this table are only slightly decreased compared to **Table 7**. This shows that the migration of the algorithm in this paper to the picking robot does not affect the accuracy of the algorithm. We also find that the values of mAP, R, and P for this paper’s algorithm are higher than the accuracy of the other six algorithms, which proves that our algorithm has the highest combined accuracy relative to the other algorithms. We continue to observe the FPS of the seven algorithms and find that the algorithm in this paper runs at a frame rate that surpasses all algorithms except YOLOv4-Tiny, reaching 47.03 FPS, but the mAP, R, and P of the algorithm in this paper are much higher than those of the other six algorithms, and the performance of the algorithm in this paper is higher than that of YOLOv4-Tiny from the perspective of comprehensive performance. In summary, the algorithm in this paper is more stable and faster on the picking robot than the other six algorithms.

**Table 8:** Comprehensive performance comparison of multiple algorithms.

Method	mAP (%)	R (%)	P (%)	FPS
1. Faster R-CNN	58.9%	61.2%	52.8%	0.25
2. SSD	58.3%	54.5%	60.9%	2.52
3. YOLOv3 (+TensorRT)	68.3%	65.4%	71.6%	27.12
4. YOLOv4 (+TensorRT)	73.5%	71.8%	76.3%	20.03
5. YOLOv4-Tiny (+TensorRT)	51.1%	55.1%	49.3%	48.97
6. YOLOv5x (+TensorRT)	68.8%	68.1%	70.8%	30.39
7. BlueberryYOLO (+TensorRT)	77.1%	75.7%	78.9%	47.06

**Figure 8:**
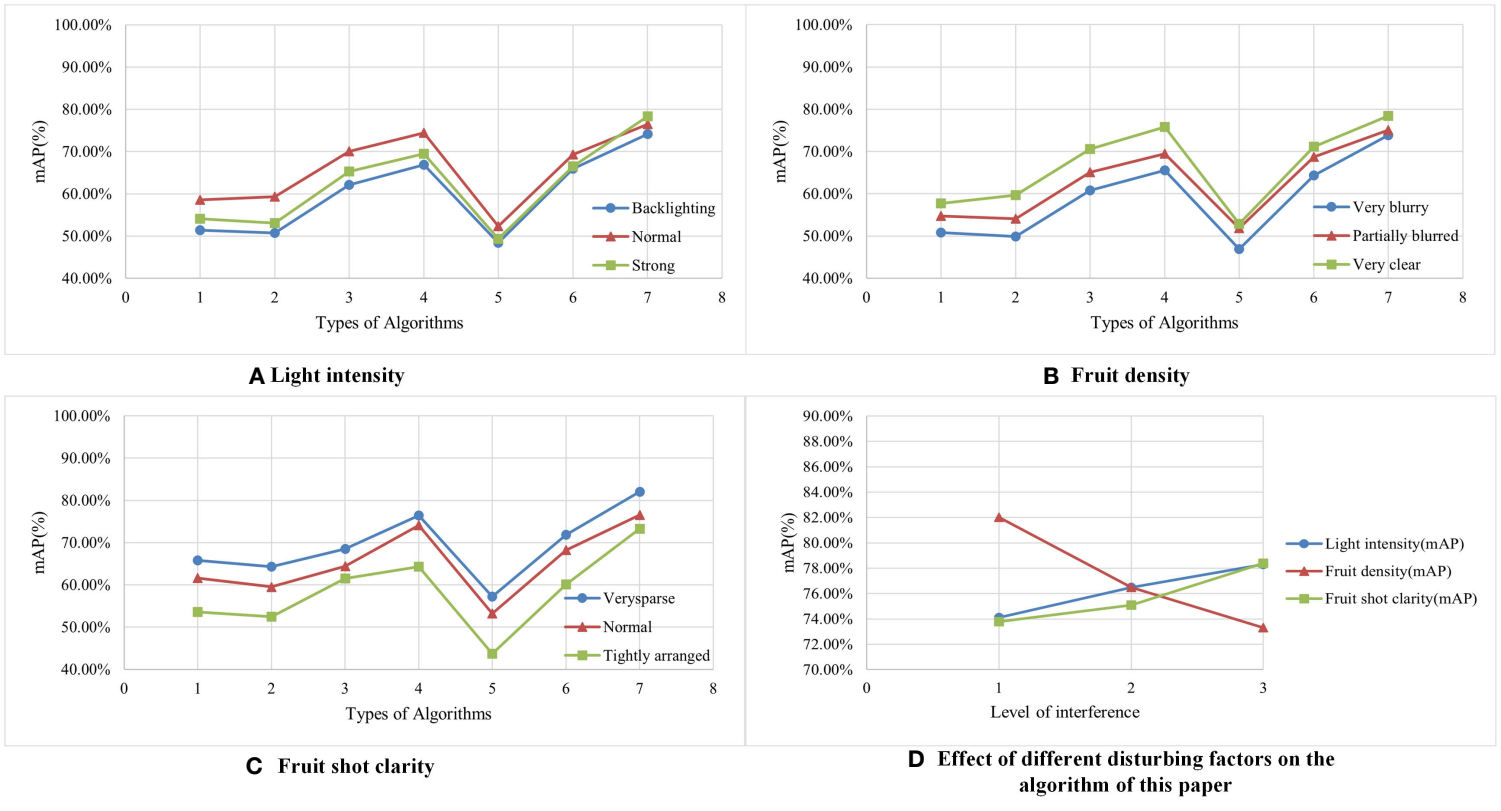
A line graph of experimental results on interference immunity of multiple algorithms **(A–C)** indicates the accuracy trends of multiple algorithms under the action of three different disturbance factors, respectively. For example, when observing the blue curve in **(D)**, 1 means Backlighting, while 2 means Normal, and 3 means Strong. When observing the green curve **(D)**, 1 means Very blurry, while 2 means Partially blurred, and 3 means Very clear. Considering that the training data set of the algorithm is from one region and only one cultivar of blueberry, we need to collect some blueberries from different regions and different cultivars of blueberries as the test set to further test the migration and applicability of the algorithm. To complete the experiment, three cultivars of blueberry were collected from three cities, namely Nanchang, Jiangxi Province, Hangzhou, Zhejiang Province, and Kunming, Yunnan Province, as test sets, namely Highbush blueberry, Lowbush blueberry, and Rabbiteye blueberry. The number distribution is shown in **Table 11**, and the test result data are shown in **Table 12**.

Although the immunity of the algorithm in this paper was initially discussed in *Section 5.5*, we do not know whether the performance of the algorithm changes after migrating it to a picking robot due to the change in the hardware environment. Therefore, we selected the corresponding set of images from the dataset of this paper to test the immunity of the algorithm applied to the picking robot. **Table 9** shows the distribution of the number of test sets used in this experiment; **Table 10** shows the experimental results data for interference immunity; and **Figure 8** shows the line graph drawn from the data in **Table 10**. Combining the data in **Table 10** and **Figures 8A–C**, we migrated all seven algorithms to the picking robot and ran them under the effect of three disturbance factors, and the final accuracy of this algorithm surpassed the other six algorithms, which indicates that this algorithm has the strongest anti-interference ability against all three disturbance factors when run on the picking robot. **Figure 8D** shows the trend of the accuracy of this algorithm for three different disturbance factors, from which we can find that the sensitivity of the accuracy of this algorithm to the density of fruit distribution will be higher than the other two disturbance factors.

**Table 9:** The test set used in this section of the immunity experiment.

Types	Number	Light intensity			Fruit density			Fruit shot clarity		
		Backlighting	Normal	Strong	Very sparse	Normal	Tightly arranged	Very blurry	Partially blurred	Very clear
Test	1,717	401	1,113	203	211	1,311	195	273	1,233	211

**Table 10:** Experimental results data of interference immunity of multiple algorithms.

Method	Light intensity (mAP)			Fruit density (mAP)			Fruit shot clarity (mAP)		
	Backlighting	Normal	Strong	Very sparse	Normal	Tightly arranged	Very blurry	Partially blurred	Very clear
1. Faster R-CNN	51.4%	58.6%	54.1%	65.8%	61.6%	53.6%	50.8%	54.7%	57.7%
2. SSD	50.7%	59.3%	53.1%	64.3%	59.5%	52.5%	49.9%	54.1%	59.7%
3. YOLOv3	62.1%	70.0%	65.3%	68.5%	64.4%	61.5%	60.8%	65.1%	70.6%
4. YOLOv4	66.9%	74.4%	69.5%	76.4%	74.1%	64.3%	65.5%	69.5%	75.8%
5. YOLOv4-Tiny	48.4%	52.3%	49.3%	57.2%	53.2%	43.7%	46.9%	51.8%	52.9%
6. YOLOv5x	65.9%	69.3%	66.5%	71.9%	68.2%	60.1%	64.3%	68.7%	71.1%
7. BlueberryYOLO	74.1%	76.5%	78.3%	82.0%	76.5%	73.3%	73.8%	75.1%	78.4%

**Table 11:** Blueberry test sets for different regions and different species.

Types	KunMing			NanChang			HangZhou		
	Highbush	Lowbush	Rabbiteye	Highbush	Lowbush	Rabbiteye	Highbush	Lowbush	Rabbiteye
**Test**	**1,311**	**1,230**	**1,453**	**1,434**	**1,331**	**1,503**	**1,453**	**1,342**	**1,591**

In analyzing the results, we need to use the column of highbush blueberries from Nanchang in **Table 12** as the control group because the dataset used for training the algorithm in this paper is from the dataset in **Table 1**, which is the highbush blueberries collected from a blueberry farm in Nanchang, Jiangxi Province.

**Table 12:** Various algorithms to identify mAP results of multiple blueberry cultivars in multiple locations.

Method	KunMing			NanChang			HangZhou		
	Highbush	Lowbush	Rabbiteye	Highbush	Lowbush	Rabbiteye	Highbush	Lowbush	Rabbiteye
1. Faster R-CNN	55.5%	53.4%	51.9%	61.7%	57.1%	55.2%	57.5%	55.3%	52.9%
2. SSD	54.7%	52.9%	52.1%	60.1%	56.5%	54.3%	56.8%	54.7%	53.5%
3. YOLOv3	66.5%	63.7%	60.1%	69.5%	65.1%	62.3%	67.1%	62.9%	59.7%
4. YOLOv4	69.2%	63.5%	66.4%	74.3%	68.8%	70.8%	70.1%	65.9%	63.2%
5. YOLOv4-Tiny	53.1%	45.7%	46.1%	56.6%	50.9%	51.4%	51.7%	43.8%	45.7%
6. YOLOv5x	64.3%	58.5%	57.4%	69.9%	63.1%	62.7%	65.3%	59.8%	58.9%
7. BlueberryYOLO	78.7%	75.7%	74.8%	79.6%	77.2%	76.9%	77.8%	75.6%	75.8%

First, we observe the three columns of Nanchang in **Table 12** separately, and we find that the accuracy of the algorithm does decrease when the recognition species of blueberry are different from the one used in the algorithm training. For example, the accuracy of the algorithm in this paper decreases from 79.6% to 77.2% and 77.8%, respectively, but the decrease is the smallest compared with the other six algorithms. Therefore, this indicates to some extent that the algorithms in this paper have better migration and adaptability to the recognition of different cultivars of blueberries. Secondly, we compare the highbush blueberries in Nanchang, Hangzhou, and Yunnan, and we can find that when the origin of the blueberries used for detection is different from the origin of the blueberries used in the training of the algorithm, the detection accuracy of the algorithm also decreases to some extent. For example, the accuracy of the algorithm in this paper decreases from 79.6% to 78.7% and 77.8%, respectively, but the decrease is minimal compared with the other six algorithms. The accuracy of this algorithm decreases from 79.6% to 78.7% and 77.8%, respectively. Therefore, to a certain extent, this experimental result can show that the algorithm in this paper has some migration and adaptability when dealing with blueberries in different cities and climates.

**Figure 9:**
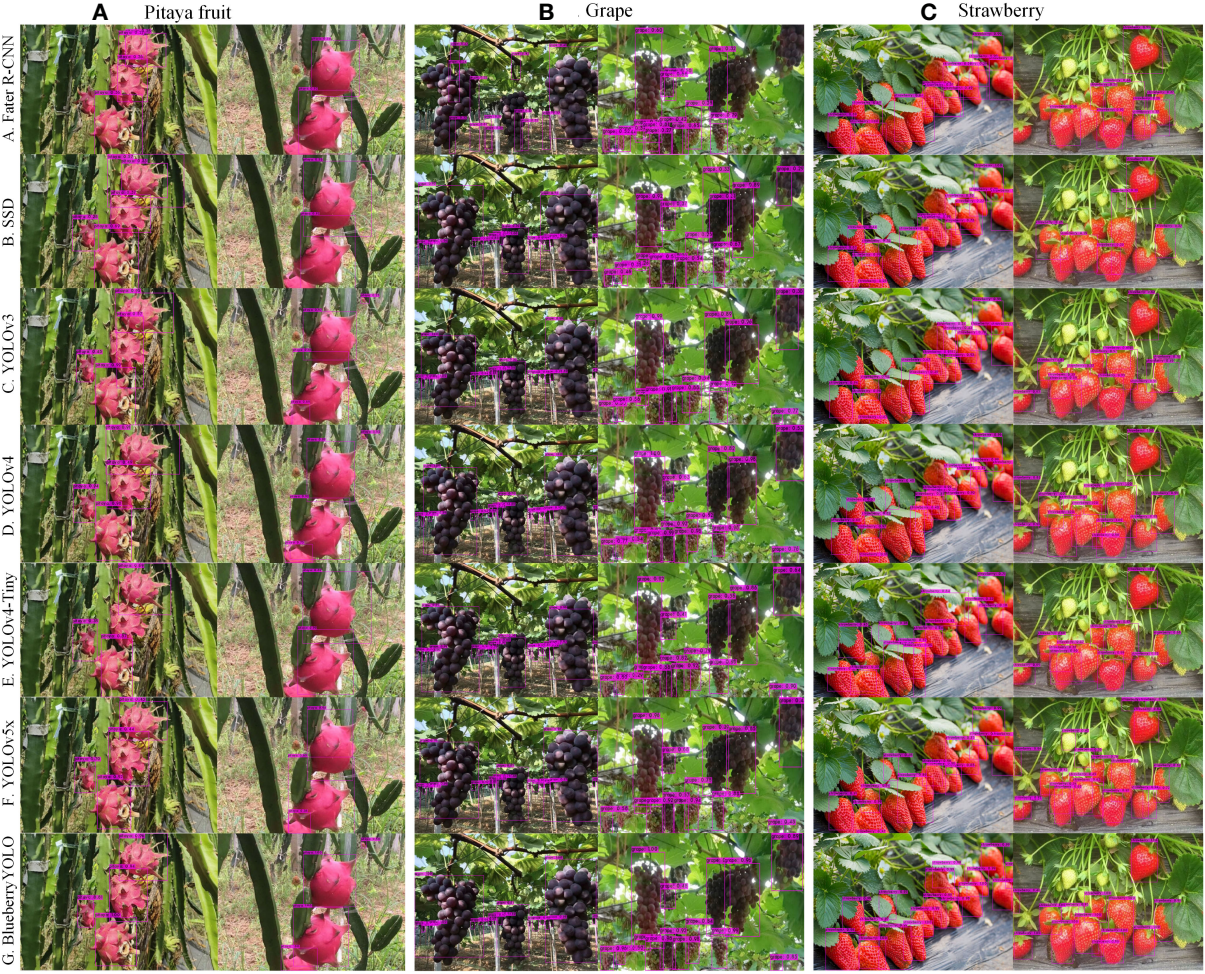
Multiple algorithms for immunity to environmental changes. **(A)** represents multiple algorithms for the detection effect of dragon fruit, **(B)** represents multiple algorithms for the detection effect of grapes, and **(C)** represents multiple algorithms for the detection effect of strawberries.

To examine the practical applicability and transferability of the algorithm to other fruits, we collected 2,000 images each of pitaya (Red Pitaya), grape (Kyoho grape), and strawberry (Red Strawberry) fruit datasets from the same origin (Nanchang) as the blueberry training set used in this paper, and then made these images into the dataset used for the final algorithm training after the data enhancement method described in *Section 2.2*. The specific number distribution of these datasets is shown in **Table 13**. We trained each of the seven algorithms directly on the above datasets to obtain recognition models for different fruits, and the final detection results are shown in **Table 14**. The detection results are shown in **Figure 9**.

**Table 13:** Number distribution of the dataset for three different cultivars of fruits.

Fruit cultivars	Number	Train	Validation	Test
**Dragon fruit**	**26,201**	**18,347**	**2,612**	**5,242**
**Grape**	**26,315**	**18,421**	**2,631**	**5,263**
**Strawberry**	**25,902**	**18,132**	**2,590**	**5,180**

**Table 14:** Multiple algorithms for different fruit detection accuracy.

Method	FPS	Pitaya fruit			Grape			Strawberry		
		AP (%)	R (%)	P (%)	AP (%)	R (%)	P (%)	AP (%)	R (%)	P (%)
1. Faster R-CNN	0.24	61.2%	63.1%	55.1%	63.3%	65.3%	58.8%	56.8%	51.3%	51.1%
2. SSD	2.49	60.1%	57.5%	62.2%	63.8%	59.9%	64.0%	56.7%	52.3%	55.1%
3. YOLOv3	27.10	70.1%	67.7%	74.1%	73.4%	70.0%	76.5%	68.3%	67.5%	69.3%
4. YOLOv4	20.02	76.2%	73.3%	78.9%	79.1%	76.5%	80.3%	73.1%	71.3%	75.7%
5. YOLOv4-Tiny	49.01	54.9%	58.3%	53.6%	57.3%	60.1%	56.3%	48.2%	45.2%	46.1%
6. YOLOv5x	30.39	71.3%	75.3%	73.2%	76.3%	74.8%	73.2%	67.4%	65.3%	68.5%
7. BlueberryYOLO	47.10	82.3%	79.4%	80.3%	84.7%	82.1%	84.1%	73.5%	74.7%	76.1%

By analyzing **Table 14**, we can see that our algorithm has achieved a good level of AP, R, and P, surpassing the other six algorithms. Our algorithm performs better at detecting grapes compared to dragon fruit and strawberries, while its detection performance for strawberries is the worst. The main reason is that the picking robot only needs to recognize a whole bunch of grapes instead of individual grapes when picking and locating them, and there is also a relatively large gap between each bunch of grapes during the actual picking process. However, strawberries are heavily obscured by leaves during their growth, which to some extent affects the accuracy of the algorithm. Overall, our algorithm has demonstrated good performance and algorithm transferability in recognizing different cultivars of fruits. The ninth figure shows the detection results of seven algorithms on three different fruits. It can be seen from this figure that the algorithm proposed in this paper has no obvious missing detection or false detection, and the confidence level of each detected target is higher than that of other algorithms.

## 6 Conclusion

In this study, we propose a BlueberryYOLO-based blueberry ripeness detection algorithm that is suitable for running on a blueberry picking robot. The experiment result shows that MobileNetv3 introduces a Little-CBAM structure, replaces the original backbone structure, enables the algorithm to have a stronger focus on blueberries, and significantly reduces the computational effort of the algorithm. This paper extends the original structure with a layer of feature fusion structure, which enables the algorithm to have a stronger feature extraction capability for small targets. We embedded a new attention mechanism, MSSENet, which can significantly enhance the feature representation capability of a small target detection network and the anti-interference capability of the algorithm. Our algorithm has a final mAP of 78.4% on the PC terminal, which far exceeds other advanced algorithms. When the algorithm was transferred to the picking robot, it was able to run at a frame rate of 47.06 FPS with no significant change in accuracy, achieving real-time operation. Also, our algorithm has good transferability and applicability, and the algorithm in this paper has the possibility of being used on other fruits as well.

## Data availability statement

The raw data supporting the conclusions of this article will be made available by the authors, without undue reservation.

## Author contributions

Conceptualization, YL and HZ. Methodology, HZ and YL. Software, HZ and YL. Validation, YZ and YL. Formal analysis, HC and QZ. Investigation, HZ and YL. Resources, YL. Data curation, YL. Writing—original draft preparation, HZ. Writing—review and editing, YZ and YL. Visualization, HZ. supervision, YL and YZ. Project administration, YL. Funding acquisition, YL. All authors listed have made a substantial, direct, and intellectual contribution to the work and approved it for publication.
